# The Modified Neo-Bioscore System for Staging Breast Cancer Treated with Neoadjuvant Therapy Based on Prognostic Significance of HER2-Low Expression

**DOI:** 10.3390/jcm13071850

**Published:** 2024-03-23

**Authors:** Yingying Zhao, Xinru Chen, Yaohui Wang, Xueqing Zhang, Jingsong Lu, Wenjin Yin

**Affiliations:** 1 Department of Breast Surgery, Renji Hospital, School of Medicine, Shanghai Jiao Tong University, No. 1630 Dongfang Road, Shanghai 200127, China; waldeinsamkeit@sjtu.edu.cn (Y.Z.); 13761501087@sjtu.edu.cn (X.C.); wangyaohui@renji.com (Y.W.); 2 Department of Pathology, Renji Hospital, School of Medicine, Shanghai Jiao Tong University, Shanghai 200127, China; zhangxueqing@renji.com

**Keywords:** HER2-low breast cancer, prognosis, Neo-Bioscore, HER2-zero, HER2-positive

## Abstract

**Background**: Recently, the classification of HER2 status evolves from binary to ternary, and HER2-low expression may exhibit prognostic significance. We aimed to investigate whether HER2-low tumor is distinct from HER2-zero or HER2-positive tumors, and then to develop a modified staging system (mNeo-Bioscore) that incorporates HER2-low status into Neo-Bioscore. **Patients and Methods**: This cohort study was conducted using data from the prospective database on breast cancer patients between January 2014 and February 2019. **Results**: Among 259 patients enrolled in the study, the HER2-low tumor exhibited significantly lower histological grade, pathological staging and Ki-67 level than the other two groups. HER2-low patients and HER2-positive patients receiving concurrent HER2-directed therapy may have similar LRFS (*p* = 0.531) and OS (*p* = 0.853), while HER2-zero peers may have significantly worse LRFS (*p* = 0.006) and OS (*p* = 0.017). In particular, a similar trend was also found in the patients without pathological complete response after surgery. Incorporation of HER2-low status made improvement in fit: 5-year OS rate estimates ranged from 33.33% to 100% for mNeo-Bioscore vs 61.36% to 100% for Neo-Bioscore. **Conclusions**: This study demonstrated that HER2-low tumor may exhibit prognostic significance. The innovative mNeo-Bioscore, based on a new classification of HER2 status, may serve as a prognostic staging system superior to Neo-Bioscore.

## 1. Introduction

Human epidermal growth factor receptor 2 (HER2) status is routinely assessed using immunohistochemistry (IHC) and/or in situ hybridization (ISH). Lately, cases with HER2 IHC 1+ or 2+ and negative ISH are classified as HER2-low breast cancer. HER2-low tumor accounts for approximately 45–55% of all breast cancers [1]. Considerable attempts have been made to investigate the prognostic value of HER2-low status. Several studies revealed a better [2,3,4,5,6,7,8,9,10] or worse [11,12] prognosis for patients with HER2-low tumor when compared to those with HER2-zero (defined as IHC 0) tumor in various settings, while others hardly found any difference between HER2-low and HER2-zero tumors [13,14,15,16,17,18,19,20]. The biological behavior of a HER2-low tumor is yet to be elucidated, especially in those administered neoadjuvant chemotherapy. Therefore, it hints at a demand to understand the distinction among HER2-zero, HER2-low and HER2-positive tumors in clinicopathological characteristics, pathological complete response (pCR) and long-term survival outcomes for locally advanced breast cancer receiving neoadjuvant chemotherapy.

Routine administration of anti-HER2 therapy such as trastuzumab has significantly altered the natural history of HER2-positive tumor, which has been incorporated in the current American Joint Committee on Cancer staging system [21]. However, few studies reported diversities between HER2-positive tumor treated with HER2-directed therapy and HER2-low or HER2-zero counterparts, especially in patients undergoing neoadjuvant chemotherapy. 

Neo-Bioscore, a newly scoring system that adds HER2 status into the previously developed CPS + EG staging system [22], can better predict long-term survival outcome in patients receiving neoadjuvant chemotherapy [23]. As the classification of HER2 status evolves from binary (negative and positive) to ternary (zero, low and positive) and HER2-low expression may serve as a unique prognosticator, whether it is appropriate to accordingly update the Neo-Bioscore scoring system or not awaits further research.

On these premises, we aimed to conduct a retrospective study based on our prospectively maintained neoadjuvant cohort to analyze the clinical significance of HER2-low status as well as to further clarify the disparities among HER2-zero, HER2-low, and HER2-positive breast cancers in patients undergoing neoadjuvant chemotherapy. We also innovatively optimized the Neo-Bioscore system according to the new classification of HER2 status (HER2-zero, HER2-low and HER2-positive), which might offer implications for subsequent clinical practice.

## 2. Patients and Methods

### 2.1. Study Design and Participants

Patients were enrolled retrospectively from the prospective database on breast cancer patients of Renji Hospital, School of Medicine, Shanghai Jiao Tong University if they were treatment-naïve females aged 18 to 70 years with histologically confirmed invasive breast cancer (T1 N1-3 or T2-4 N0-3, M0) and available HER2 status from January 2014 to February 2019. We excluded patients if they were in pregnancy or lactation, had metastatic or bilateral breast cancer, had a history of malignancy other than breast cancer, or HER2-positive patients without application of anti-HER2 targeted therapy. All patients received weekly paclitaxel-based chemotherapy in the neoadjuvant setting and underwent surgery as planned after neoadjuvant treatment. For HER2-positive patients, trastuzumab was recommended concurrently at a loading dose of 4 mg/kg followed by a maintenance dose of 2 mg/kg weekly thereafter. Adjuvant treatment was tailored after surgery according to the guidelines at that time or at the discretion of physicians.

The protocol was approved by the Independent Ethics Committee of Renji Hospital (Approval number: LY2022-028-B, date: 28 October 2022). This study was registered with ClinicalTrials.gov (NCT05621564). 

### 2.2. Clinicopathological Parameters

The baseline clinicopathological parameters were prospectively collected, including age, menopausal status, clinical stage, as well as estrogen receptor (ER), progesterone receptor (PR), HER2 status, Ki-67 levels, and histological grade before neoadjuvant treatment. The initial evaluation comprised a complete medical history, physical examination, electrocardiogram, chest computed tomography scan, ultrasound of breast/regional lymph nodes/abdomen, bilateral mammography, breast magnetic resonance imaging and positron emission tomography if necessary. During neoadjuvant therapy, breast ultrasound and magnetic resonance imaging were examined every two cycles for the assessment of lesion changes according to the Response Evaluation Criteria in Solid Tumors (RECIST) version 1.1. The pathological information and follow-up data were also recorded prospectively. Outpatient visits or telephone interviews occurred every 3 months in the first 2 years after surgery. Thereafter, follow-up was advised every 6 months until the fifth year and then annually until death or any relapse.

All the biopsy tissues were confirmed as invasive breast cancer at the Department of Pathology, Renji Hospital. Tumors were considered hormone receptor-positive if at least 1% of invasive tumor cells exhibited immunostaining for ER or PR according to the American Society of Clinical Oncology/College of American Pathologists (ASCO/CAP) guideline recommendations for ER and PR testing [24]. HER2 positivity was defined as IHC 3+ or IHC 2+ with amplification confirmed by fluorescent in situ hybridization (FISH) based on the ASCO/CAP 2018 guideline [25]. As per the most recent consensus [1,26], HER2-negative tumors were divided into two groups: HER2-zero for tumors scored IHC 0 and HER2-low for tumors scored IHC 1+ or 2+ with a nonamplified FISH assay.

### 2.3. Neo-Bioscore and mNeo-Bioscore Staging Systems

Neo-Bioscore is a staging system using clinical stage, response to therapy, and biological subtype, proposed recently to determine the outcome of patients with breast cancer receiving neoadjuvant treatment. It is determined according to the previously published scoring principle [23] (Table 1). In brief, scoring points are assigned by presenting the clinical stage, final pathological stage, and the biomarkers, including ER (1% as the cutoff for ER positivity), grade, and HER2 status. The modified staging system, entitled mNeo-Bioscore, was put forward based on our data, in which HER2 status was assigned 1 point for HER2-zero patients, and 0 points for both HER2-low tumor and HER2-positive counterparts treated with anti-HER2 agents; the rest of the assignment principles remained unchanged. The points were added up to determine a Neo-Bioscore or mNeo-Bioscore score.

### 2.4. Statistical Analyses

Correlations between HER2 status and clinicopathological parameters were evaluated using Fisher’s exact or χ^2^ test for categorical variables. The continuous variables were analyzed using Wilcoxon or Kruskal–Wallis tests where appropriate. 

Study outcomes were pathologic complete response (pCR included pCR_T0_ and pCR_T0/is_) and different survival outcomes. We defined pCR_T0_ and pCR_T0/is_ as ypT0 ypN0 and ypT0/is ypN0, respectively. The association between HER2 status and pCR was tested by logistic regression models. Odds ratios (ORs) with 95% confidence intervals (CIs) were derived. Locoregional relapse-free survival (LRFS) was estimated from surgery to the first occurrence of locoregional relapse, or death, regardless of cause. Overall survival (OS) denoted the time from surgery to death, irrespective of cause. Disease-free survival (DFS), relapse-free survival (RFS), and distant relapse-free survival (DRFS) were also analyzed. Kaplan–Meier curves and log-rank tests were used to compare results among groups. Multivariate Cox proportional hazard models were fitted to examine the prognostic value by reporting hazard ratios (HRs) with 95% CIs. All multivariate analyses included age, HER2 status, hormone receptor status, Ki-67 index level, histological grade, clinical T stage, and clinical N stage. Two extra covariates, pathological T stage and pathological N stage, were added to models for survival outcomes. 

Fits of the Cox proportional hazard model for Neo-Bioscore and mNeo-Bioscore systems were compared by the Akaike Information Criterion (AIC). The receiver operating characteristic (ROC) curve, concordance index (C-index), and integrated discrimination improvement (IDI) were employed to identify prediction accuracy. Decision curve analysis (DCA) was utilized to evaluate clinical benefit of two scoring systems. Statistical tests were by default two-sided with a significance level of 0.05. Bonferroni correction was put to use for multiple testing. All statistical analyses were performed by RStudio v4.1.1 http://www.R-project.org (accessed on 4 December 2022).

## 3. Results

### 3.1. Patient Characteristics

Between January 2014 and February 2019, 280 patients with locally advanced invasive breast cancer were screened and 259 eligible patients were enrolled in the analysis, consisting of 155 HER2-negative (66 HER2-zero and 89 HER2-low) and 104 HER2-positive patients (Supplementary Figure S1). The median follow-up time was 5.74 years.

HER2-low tumors occupied 34.4% of all the enrolled patients. Hormone receptor positivity was numerically more common in HER2-low breast cancer than in the other two groups (83.2% in HER2-low vs 72.7% in HER2-zero vs 77.9% in HER2-positive, *p* = 0.293; Supplementary Figure S2). A significant difference among the three groups was detected for Ki-67 index, with 66.3% exhibiting Ki-67 expression > 20% in HER2-low tumor, compared with 80.3% in HER2-zero and 81.7% in HER2-positive tumors (*p* = 0.028; Table 2). Besides, statistically significant differences were also observed in terms of histological grade (*p* = 0.005; Table 2), pathological T stage (*p* = 0.005; Table 2), pathological N stage (*p* < 0.001; Table 2), and pathological staging (*p* < 0.001; Table 2). 

### 3.2. Association of HER2 Status with pCR

We compared the pCR_T0_ and pCR_T0/is_ rate among HER2-zero, HER2-low and HER2-positive tumors, respectively (Supplementary Figure S3). HER2-positive patients treated with target therapy had significantly higher pCR rates compared with HER2-low (pCR_T0_, OR, 3.619 [95% CI, 1.861–7.037]; *p* < 0.001) and HER2-zero counterparts (pCR_T0_, OR, 2.478 [95%CI, 1.252–4.907]; *p* = 0.009) respectively in the entire population (Supplementary Figure S3A). A similar trend was also found in the hormone receptor-positive subgroup (Supplementary Figure S3A). Likewise, the pCR_T0/is_ rate showed the same pattern as the pCR_T0_ rate (Supplementary Figure S3B). The multivariate logistic regression analysis indicated that HER2 status (HER2-positive vs. HER2-low and HER2-positive vs HER2-zero) was an independent predictor for pCR (pCR_T0_, HER2-positive vs HER2-low: OR, 4.263 [95%CI, 1.889–9.621], *p* < 0.001; HER2-positive vs. HER2-zero: OR, 3.195 [95%CI, 1.421–7.182], *p* = 0.005). However, no significant differences in the pCR_T0_ and pCR_T0/is_ rates were found between HER2-low and HER2-zero tumors in both univariate and multivariate analyses. 

### 3.3. Association of HER2 Status with Survival Outcomes

**Figure 1:**
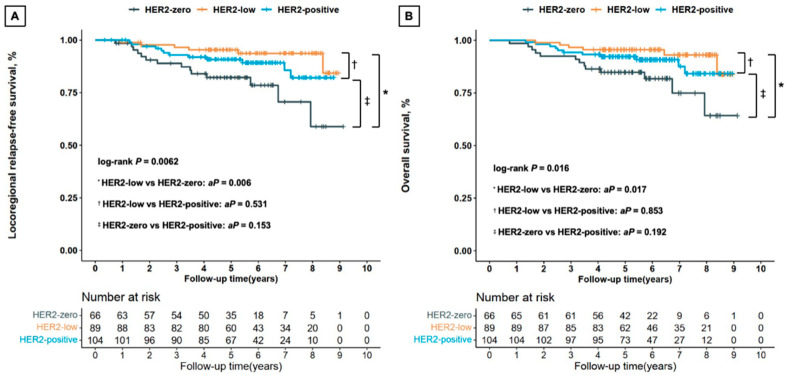
Kaplan–Meier survival analysis by HER2 status for locoregional relapse-free survival (**A**) and overall survival (**B**) in all the enrolled patients. Abbreviations: HER2—human epidermal growth factor receptor 2; *aP*—*p*-value adjusted by Bonferroni correction in all the enrolled patients.

Kaplan–Meier curves showed that HER2-low breast cancers had significantly better LRFS than HER2-zero tumors (Bonferroni corrected *p* = 0.006), but there was no statistically significant difference between the HER2-low and HER2-positive tumors (Bonferroni corrected *p* = 0.531) or between the HER2-zero and HER2-positive tumors (Bonferroni corrected *p* = 0.153; Figure 1A). Multivariate Cox regression analysis indicated superior LRFS for HER2-low patients compared with HER2-zero women (HR, 0.207 [95%CI, 0.070–0.610]; *p* = 0.004), and no statistically remarkable distinction between HER2-low and HER2-positive tumors (HR, 0.385 [95%CI, 0.137–1.085]; *p* = 0.071). In the hormone receptor-positive subgroup, HER2-low tumor had longer LRFS than HER2-zero tumor (univariate analysis, Bonferroni corrected *p* = 0.053, Supplementary Figure S4A; multivariate analysis, *p* = 0.023).

The OS was remarkably different among HER2-zero, HER2-low, and HER2-positive patients (Figure 1B). Multivariate Cox regression analysis revealed that HER2-low tumor was significantly related to longer OS outcomes compared with HER2-zero tumor (HR, 0.258 [95%CI, 0.087–0.765]; *p* = 0.015, Supplementary Figure S5), while there was no significant difference between HER2-low and HER2-positive tumors (HR, 0.425 [95%CI, 0.146–1.230]; *p* = 0.115). Interestingly, HER2-zero breast cancers had worse OS outcomes compared with HER2-positive peers in the hormone receptor-negative patients (univariate analysis, Bonferroni corrected *p* = 0.080, Supplementary Figure S4B; multivariate analysis, *p* < 0.001). 

In contrast, no significant differences were seen in terms of DFS, RFS, and DRFS among HER2-zero, HER2-low, and HER2-positive tumors for all the enrolled patients as well as subgroups by hormone receptor status. 

### 3.4. Effect of pCR Status on Survival Outcomes by HER2 Status 

**Figure 2:**
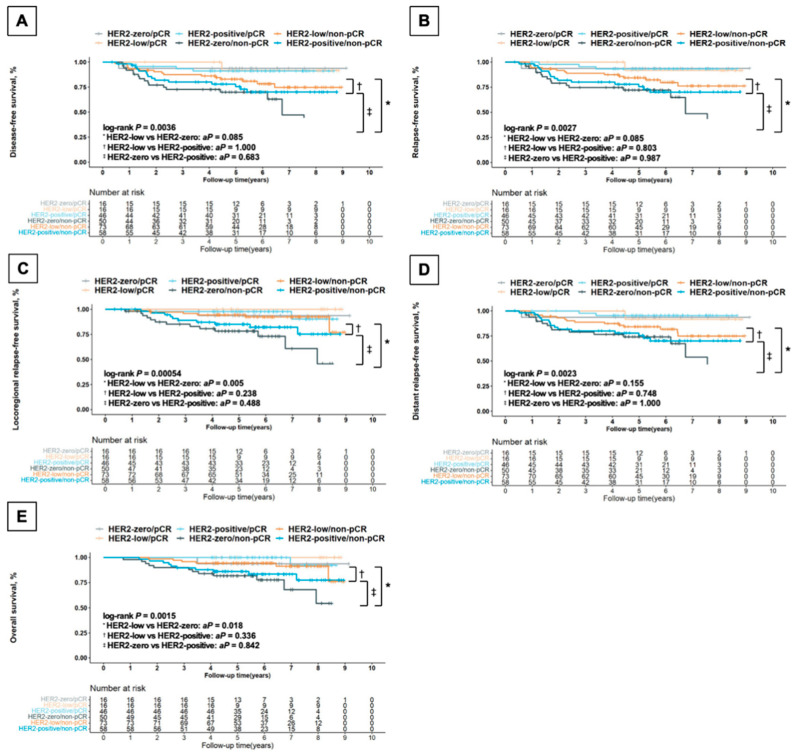
Kaplan–Meier survival analysis by HER2 status and postoperative pathological status for disease-free survival (**A**), relapse-free survival (**B**), locoregional relapse-free survival (**C**), distant relapse-free survival (**D**), and overall survival (**E**) in all the enrolled patients. Abbreviations: HER2—human epidermal growth factor receptor 2; pCR—pathological complete response, defined as ypT0 ypN0; *aP*—*p*-value adjusted by Bonferroni correction in the non-pCR patients.

According to different combinations of postoperative pathological status (pCR/non-pCR) and HER2 status, subgroups obviously differed in terms of various survival outcomes (Figure 2). As for those with non-pCR, the HER2-low subgroup exhibited remarkably better LRFS (Bonferroni corrected *p* = 0.005, Figure 2C) and OS (Bonferroni corrected *p* = 0.018, Figure 2E) compared with HER2-zero patients. Multivariate Cox regression analysis in the non-pCR patients revealed better LRFS (HR, 0.260 [95%CI, 0.087–0.779]; *p* = 0.016) and OS (HR, 0.328 [95%CI, 0.109–0.990]; *p* = 0.048) for HER2-low patients compared with HER2-zero women, while no remarkable distinction was observed between HER2-low and HER2-positive tumors (LRFS, HR, 0.388 [95%CI, 0.135–1.112], *p* = 0.078; OS, HR, 0.419 [95%CI, 0.145–1.213], *p* = 0.109). When it came to the pCR counterparts, there was no statistically significant difference among HER2-zero, HER2-low and HER2-positive tumors in terms of OS and LRFS for either univariate or multivariate analysis.

### 3.5. Establishment of mNeo-Bioscore System 

We assigned scores to 251 patients based on Neo-Bioscore and mNeo-Bioscore systems, respectively (Supplementary Table S2). The AIC value of mNeo-Bioscore was smaller than Neo-Bioscore in predicting prognosis, indicating that mNeo-Bioscore model fitted slightly better (Supplementary Table S3). ROC curves were compared separately, and mNeo-Bioscore had a larger area under the curve than Neo-Bioscore in terms of various survival outcomes (Supplementary Figure S6). Besides, C-index (Supplementary Table S4) and IDI (Supplementary Table S5) were also estimated, indicating that mNeo-Bioscore predicted prognosis with greater accuracy than Neo-Bioscore. Furthermore, DCA showed that mNeo-Bioscore was clinically useful and had a better discriminative ability to recognize patients at high risk than Neo-Bioscore (Supplementary Figure S7). 

**Figure 3:**
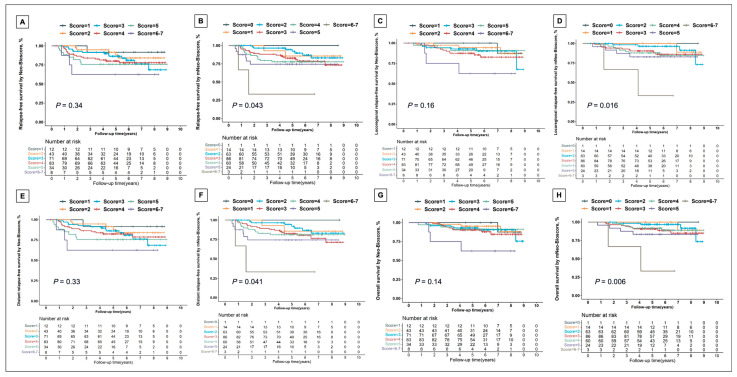
Kaplan–Meier survival analysis for relapse-free survival (**A**,**B**), locoregional relapse-free survival (**C**,**D**), distant relapse-free survival (**E**,**F**), and overall survival (**G**,**H**) by Neo-Bioscore (**A**,**C**,**E**,**G**) and mNeo-Bioscore (**B**,**D**,**F**,**H**).

We analyzed Kaplan–Meier curves with different scores according to Neo-Bioscore or mNeo-Bioscore system, which showed that mNeo-Bioscore was more capable of stratifying patients with respect to RFS (Figure 3A,B), LRFS (Figure 3C,D), DRFS (Figure 3E,F), and OS (Figure 3G,H) than Neo-Bioscore system. We found significant improvement in fit: 5-year OS rate estimates ranged from 33.33% to 100% for mNeo-Bioscore vs 61.36% to 100% for Neo-Bioscore (Supplementary Table S6).

## 4. Discussion

Our study detected differences among HER2-zero, HER2-low and HER2-positive tumors, focusing on patients who received neoadjuvant chemotherapy. It filled gaps in this field and suggested that HER2-low tumor might be associated with favorable prognosis, which was better than HER2-zero tumor and similar to HER2-positive tumor receiving anti-HER2 target therapy. We also proposed mNeo-Bioscore, an optimized staging system based on Neo-Bioscore, by altering the scoring points of HER2-low tumor, which allowed for the precise prognosis stratification of patients with breast cancer after neoadjuvant therapy.

Our data showed that HER2-low tumor accounted for approximately 57.42% of all HER2-negative tumors. The histological grade, pathological T stage, pathological N stage, and pathological staging were significantly distinct among HER2-zero, HER2-low and HER2-positive tumors, indicating that clinicopathological characteristics may appear to differ among these groups. Similar to previous studies [2,6], the Ki-67 level in HER2-low tumor was significantly lower than that in HER2-zero or HER2-positive tumors (median Ki67 index, 30% in HER2-low vs 50% in HER2-zero vs 40% in HER2-positive tumors, *p* = 0.010), suggesting a slower proliferation rate of HER2-low breast cancer. These findings imply that a HER2-low tumor may be less malignant than HER2-zero or HER2-positive tumors. 

Our study indicated that long-term survival was superior for HER2-low patients treated with neoadjuvant chemotherapy to that for HER2-zero counterparts. A pooled analysis of four prospective neoadjuvant clinical trials [2] and another cohort study using data from a prospectively maintained institutional database [14] described significantly longer DFS and OS for patients with HER2-low tumor than that for patients with HER2-zero tumor. Additionally, Zhang et al. found a significantly lower proportion of relapsed/progressed patients in the HER2-low subgroup than the HER2-zero subgroup across follow-up time points [6]. All these findings indicated that women with a HER2-low tumor are expected to have superior survival outcomes than those with a HER2-zero tumor, which was concordant with our analysis. 

Furthermore, our data showed significantly prolonged OS for HER2-low breast cancer compared with HER2-zero counterparts, even when adjusted for hormone receptor status. In line with our study, a large cohort study conducted by Peiffer et al. with a longer median follow-up time of 54 months identified substantial improvement in OS favoring HER2-low patients for stages II to IV triple-negative breast cancer and stages III to IV hormone receptor-positive/HER2-negative disease when analyzed by hormone receptor status and clinical stage [27]. Intriguingly, Tarantino and colleagues found no significant distinction in prognosis between HER2-low and HER2-zero tumors when adjusting for confounders including hormone receptor status [14]. However, the majority of patients included in their study were early-stage breast cancer (stage I, 72%) and the median follow-up time of 10 months may seem insufficient for survival analysis, which was largely different from those of the studies by Peiffer et al. and our group. 

Few studies have been reported with regard to the differences between HER2-negative and HER2-positive tumors treated with neoadjuvant chemotherapy plus trastuzumab. The NOAH study [28] showed that neoadjuvant anti-HER2 targeted therapy resulted in comparable prognoses between HER2-positive and HER2-negative tumors, but did not distinguish HER2-low and HER2-zero subgroups. We first detected a significantly higher pCR rate in HER2-positive patients treated with neoadjuvant chemotherapy combined with trastuzumab than that in HER2-low patients receiving neoadjuvant chemotherapy. Nevertheless, they had similar survival outcomes. On the other hand, the HER2-positive tumors achieved significantly higher pCR rates than HER2-zero peers in the whole group, and translated into OS benefit in the hormone receptor-negative subgroup. Notably, the study by Zhang et al., which enrolled mostly early-stage breast cancers (72.3%) and even a small portion of metastatic breast cancers (5.0%), revealed significantly longer DFS for HER2-positive breast cancers treated with trastuzumab than that for HER2-zero breast cancers [6], partially supporting our findings. 

Sequential adjuvant treatment for patients with non-pCR after neoadjuvant therapy is one of the hot and key clinical issues. Our data suggested that both pCR and HER2 status affected survival outcomes for breast cancer. Thereby, the delineation of HER2-positive, HER2-low and HER2-zero tumors can contribute to the precise stratification and personalized treatment of non-pCR patients. As we all know, based on current guidelines, extended adjuvant chemotherapy or specific targeted therapy is recommended after surgery for HER2-negative or HER2-positive patients without pCR. With the future application of antibody-drug conjugates (ADCs) [29,30,31], such as trastuzumab deruxtecan, gradually moving forward to (neo)adjuvant therapy (NCT04622319), it is worth further exploring whether existing treatments for HER2-low/non-pCR patients can be replaced with such ADCs, due to their similar survival to HER2-positive/non-pCR peers. On the other hand, the escalation of currently recommended adjuvant treatment [32,33,34], might be even more necessary for HER2-zero/non-pCR patients due to their worse prognosis compared with HER2-low/non-pCR counterparts. Further trials are warranted to optimize the strategy of HER2-low/non-pCR and HER2-zero/non-pCR tumors.

With the increasing use of neoadjuvant targeted therapy for HER2-positive tumor, the prognostic value of CPS + EG staging system [22] was partly compromised due to favorable response of HER2-positive tumor to trastuzumab combined with chemotherapy, leading to the birth of the Neo-Bioscore that significantly improved the predictive performance of 5-year disease-specific survival (DSS) compared to the CPS + EG staging system [23]. Additionally, further understanding of HER2 status may result in the inevitable evolution of Neo-Bioscore. On the basis of our study, the prognosis for a HER2-low tumor is significantly better than that for a HER2-zero tumor and similar to that for HER2-positive patients receiving targeted therapy, not only in the whole group (despite their relatively different pCR rates) but also in the non-pCR subgroup. Therefore, it is a reasonable attempt to assign a score of 0 to either the HER2-low tumor or the HER2-positive tumor treated with targeted therapy, and 1 to HER2-zero breast cancers in the mNeo-Bioscore system. Moreover, a series of multidimensional validations confirmed our idea that the mNeo-Bioscore may fit better, and is capable of accurately predicting various survival outcomes in patients receiving neoadjuvant treatment. The mNeo-Bioscore system better stratified patients in terms of OS, with 5-year OS estimates ranging from 33.33% to 100%. In short, the mNeo-Bioscore staging system merits further substantiation to lay the foundation for its extrapolation.

This study has some limitations. Firstly, although all data of the patients were collected prospectively, it is a retrospective analysis. Secondly, the sample of patients is not large enough. However, it is undeniable that the potential patterns discovered through exploratory analysis may help guide subsequent prospective studies. Last but not least, the median follow-up time of 5.74 years in our study may be insufficient. The long-term survival significance among HER2-zero, HER2-low and HER2-positive tumors will be further elucidated with a longer follow-up.

In conclusion, our study demonstrated that HER2-low breast cancers may exhibit prognostic significance, providing heuristic insights into the current landscape of patients receiving neoadjuvant treatment. The innovative mNeo-Bioscore, based on the new classification of HER2 status, may serve as an optimized prognostic staging system superior to Neo-Bioscore.

## Figures and Tables

**Table 1:** Point Assignments for the Neo-Bioscore and mNeo-Bioscore Staging Systems.

Cancer Stage	**Neo-Bioscore Points**	**mNeo-Bioscore Points**
Clinical stage		
I-IIA	0	0
IIB-IIIA	1	1
IIIB-IIIC	2	2
Pathological stage		
0-I	0	0
IIA-IIIB	1	1
IIIC	2	2
Tumor marker		
ER-negative	1	1
Grade 3	1	1
HER2 status		
HER2-zero	1	1
HER2-low	1	0
HER2-positive	0	0

Abbreviations: ER—estrogen receptor; HER2—human epidermal growth factor receptor 2.

**Table 2:** Clinicopathological differences in patients with different HER2 status.

	HER2-Zero (*n* = 66)	HER2-Low (*n* = 89)	HER2-Positive (*n* = 104)	*p*-Value
Age, years				
median (IQR)	48 (41, 60)	52 (43, 59)	53 (46, 60)	0.180
>50	28 (42.4%)	47 (52.8%)	63 (60.6%)	0.069
≤50	38 (57.6%)	42 (47.2%)	41 (39.4%)	
Hormone receptor status				
Negative	18 (27.3%)	15 (16.9%)	23 (22.1%)	0.293
Positive	48 (72.7%)	74 (83.1%)	81 (77.9%)	
Ki-67 index				
median (IQR)	50 (30, 70)	30 (20, 50)	40 (30, 60)	0.010
>20%	53 (80.3%)	59 (66.3%)	85 (81.7%)	0.028
≤20%	13 (19.7%)	30 (33.7%)	19 (18.3%)	
Histological grade				
Grade II	30 (48.4%)	44 (51.8%)	31 (29.8%)	0.005
Grade III	32 (51.6%)	41 (48.2%)	73 (70.2%)	
NA ^a^	4	4	0	
Clinical T stage				
cT1	0 (0%)	0 (0%)	2 (1.9%)	0.451
cT2	15 (22.7%)	17 (19.1%)	22 (21.1%)	
cT3	36 (54.6%)	41 (46.1%)	45 (43.3%)	
cT4	15 (22.7%)	31 (34.8%)	35 (33.7%)	
Clinical N stage				
cN0	9 (13.6%)	14 (15.7%)	13 (12.5%)	0.671
cN1	45 (68.2%)	62 (69.7%)	79 (76.0%)	
cN2	2 (3.0%)	3 (3.4%)	5 (4.8%)	
cN3	10 (15.2%)	10 (11.2%)	7 (6.7%)	
Clinical staging				
IIA	1 (1.5%)	1 (1.1%)	3 (2.9%)	0.552
IIB	18 (27.3%)	23 (25.9%)	29 (27.9%)	
IIIA	25 (37.9%)	29 (32.6%)	33 (31.7%)	
IIIB	12 (18.2%)	26 (29.2%)	32 (30.8%)	
IIIC	10 (15.1%)	10 (11.2%)	7 (6.7%)	
Pathological T stage				
ypT0	24 (36.4%)	29 (32.6%)	63 (60.6%)	0.005
ypTis	1 (1.5%)	4 (4.5%)	3 (2.9%)	
ypT1	31 (47.0%)	36 (40.5%)	27 (26.0%)	
ypT2	9 (13.6%)	19 (21.3%)	9 (8.6%)	
ypT3	1 (1.5%)	1 (1.1%)	2 (1.9%)	
Pathological N stage				
ypN0	33 (50.0%)	37 (41.6%)	78 (75.0%)	<0.001
ypN1	18 (27.3%)	27 (30.3%)	15 (14.4%)	
ypN2	5 (7.6%)	13 (14.6%)	8 (7.7%)	
ypN3	10 (15.1%)	12 (13.5%)	3 (2.9%)	
Pathological staging				
0	17 (25.8%)	20 (22.5%)	49 (47.1%)	<0.001
I A	12 (18.2%)	11 (12.3%)	26 (25.0%)	
II A	19 (28.8%)	25 (28.1%)	15 (14.4%)	
II B	2 (3.0%)	8 (9.0%)	3 (2.9%)	
III A	6 (9.1%)	13 (14.6%)	8 (7.7%)	
III C	10 (15.1%)	12 (13.5%)	3 (2.9%)	

Abbreviations: HER2—human epidermal growth factor receptor 2; IQR—interquartile range; NA—not applicable. ^a^ Histological grade could not be assessed in eight patients.

## Data Availability

The datasets used and/or analyzed during the current study are available from the corresponding authors on reasonable request.

## Supplementary Materials

The following supporting information can be downloaded at: https://www.mdpi.com/article/10.3390/jcm13071850/s1, Figure S1: Diagram of the study design; Figure S2: Classification of breast cancer based on different combinations of hormone receptor and HER2 status; Figure S3: Rates of pathological complete response ypT0 ypN0 (A) and ypT0/is ypN0 (B) by HER2 status in all the enrolled patients as well as subgroups by hormone receptor status; Figure S4: Kaplan-Meier survival analysis by HER2 status for locoregional relapse-free survival in the hormone receptor-positive patients (A) and overall survival in the hormone receptor-negative patients (B); Figure S5: Multivariate Cox regression analysis for overall survival in all the enrolled patients; Figure S6: Receiver Operating Characteristic curve of Neo-Bioscore (A,C,E,G,I) and mNeo-Bioscore (B,D,F,H,J) for disease-free survival (A,B), relapse-free survival (C,D), locoregional relapse-free survival (E,F), distant relapse-free survival (G,H), and overall survival (I,J) in all the enrolled patients; Figure S7: Decision curve analysis of Neo-Bioscore and mNeo-Bioscore for disease-free survival (A), relapse-free survival (B), locoregional relapse-free survival (C), distant relapse-free survival (D), and overall survival (E) in all the enrolled patients; Table S1: Clinicopathological differences in patients with different HER2 status; Table S2: Changes on the number of patients according to different scoring principles of Neo-Bioscore and mNeo-Bioscore systems; Table S3: Akaike Information Criterion value of Neo-Bioscore and mNeo-Bioscore for disease-free survival, relapse-free survival, locoregional relapse-free survival, distant relapse-free survival, and overall survival in all the enrolled patients; Table S4: Concordance index value of Neo-Bioscore and mNeo-Bioscore for disease-free survival, relapse-free survival, locoregional relapse-free survival, distant relapse-free survival, and overall survival in all the enrolled patients; Table S5: Integrated Discrimination Improvement value of mNeo-Bioscore compared with Neo-Bioscore for disease-free survival, relapse-free survival, locoregional relapse-free survival, distant relapse-free survival, and overall survival in all the enrolled patients during follow-up interval; Table S6: Five-Year overall survival rate by Neo-Bioscore and mNeo-Bioscore.

## References

[1] Tarantino P., Hamilton E., Tolaney S.M., Cortes J., Morganti S., Ferraro E., Marra A., Viale G., Trapani D., Cardoso F. (2020). HER2-Low Breast Cancer: Pathological and Clinical Landscape. J. Clin. Oncol..

[2] Denkert C., Seither F., Schneeweiss A., Link T., Blohmer J.-U., Just M., Wimberger P., Forberger A., Tesch H., Jackisch C. (2021). Clinical and molecular characteristics of HER2-low-positive breast cancer: Pooled analysis of individual patient data from four prospective, neoadjuvant clinical trials. Lancet Oncol..

[3] Almstedt K., Heimes A.-S., Kappenberg F., Battista M.J., Lehr H.-A., Krajnak S., Lebrecht A., Gehrmann M., Stewen K., Brenner W. (2022). Long-term prognostic significance of HER2-low and HER2-zero in node-negative breast cancer. Eur. J. Cancer.

[4] Kang S., Lee S.H., Lee H.J., Jeong H., Jeong J.H., Kim J.E., Ahn J.-H., Jung K.H., Gong G., Kim H.H. (2022). Pathological complete response, long-term outcomes, and recurrence patterns in HER2-low versus HER2-zero breast cancer after neoadjuvant chemotherapy. Eur. J. Cancer.

[5] Mutai R., Barkan T., Moore A., Sarfaty M., Shochat T., Yerushalmi R., Stemmer S.M., Goldvaser H. (2021). Prognostic impact of HER2-low expression in hormone receptor positive early breast cancer. Breast.

[6] Zhang G., Ren C., Li C., Wang Y., Chen B., Wen L., Jia M., Li K., Mok H., Cao L. (2022). Distinct clinical and somatic mutational features of breast tumors with high-, low-, or non-expressing human epidermal growth factor receptor 2 status. BMC Med..

[7] Li Y., Abudureheiyimu N., Mo H., Guan X., Lin S., Wang Z., Chen Y., Chen S., Li Q., Cai R. (2022). In Real Life, Low-Level HER2 Expression May Be Associated with Better Outcome in HER2-Negative Breast Cancer: A Study of the National Cancer Center, China. Front. Oncol..

[8] Xu H., Han Y., Wu Y., Wang Y., Li Q., Zhang P., Yuan P., Luo Y., Fan Y., Chen S. (2022). Clinicopathological Characteristics and Prognosis of HER2-Low Early-Stage Breast Cancer: A Sin-gle-Institution Experience. Front. Oncol..

[9] Zhou S., Liu T., Kuang X., Zhen T., Shi H., Lin Y., Shao N. (2023). Comparison of clinicopathological characteristics and response to neoadjuvant chemotherapy between HER2-low and HER2-zero breast cancer. Breast.

[10] Li J.-J., Yu Y., Ge J. (2023). HER2-low-positive and response to NACT and prognosis in HER2-negative non-metastatic BC. Breast Cancer.

[11] Zhang S., Liu Y., Liu X., Liu Y., Zhang J. (2023). Prognoses of Patients with Hormone Receptor-Positive and Human Epidermal Growth Factor Receptor 2-Negative Breast Cancer Receiving Neoadjuvant Chemotherapy before Surgery: A Retrospective Analysis. Cancers.

[12] Gilcrease M.Z., Woodward W.A., Nicolas M.M., Corley L.J., Fuller G.N., Esteva F.J., Tucker S.L., Buchholz T.A. (2009). Even low-level HER2 expression may be associated with worse outcome in node-positive breast cancer. Am. J. Surg. Pathol..

[13] Hein A., Hartkopf A.D., Emons J., Lux M.P., Volz B., Taran F.-A., Overkamp F., Hadji P., Tesch H., Häberle L. (2021). Prognostic effect of low-level HER2 expression in patients with clinically negative HER2 status. Eur. J. Cancer.

[14] Tarantino P., Jin Q., Tayob N., Jeselsohn R.M., Schnitt S.J., Vincuilla J., Parker T., Tyekucheva S., Li T., Lin N.U. (2022). Prognostic and Biologic Significance of ERBB2-Low Expression in Early-Stage Breast Cancer. JAMA Oncol..

[15] Jacot W., Maran-Gonzalez A., Massol O., Sorbs C., Mollevi C., Guiu S., Boissière-Michot F., Ramos J. (2021). Prognostic Value of HER2-Low Expression in Non-Metastatic Triple-Negative Breast Cancer and Correlation with Other Biomarkers. Cancers.

[16] Tarantino P., Niman S.M., Erick T.K., Priedigkeit N., Harrison B.T., Giordano A., Nakhlis F., Bellon J.R., Parker T., Strauss S. (2022). HER2-low inflammatory breast cancer: Clinicopathologic features and prognostic implications. Eur. J. Cancer.

[17] Chen M., Chen W., Liu D., Chen W., Shen K., Wu J., Zhu L. (2022). Prognostic values of clinical and molecular features in HER2 low-breast cancer with hormonal receptor overexpression: Features of HER2-low breast cancer. Breast Cancer.

[18] Shao Y., Yu Y., Luo Z., Guan H., Zhu F., He Y., Chen Q., Liu C., Nie B., Liu H. (2022). Clinical, Pathological Complete Response, and Prognosis Characteristics of HER2-Low Breast Cancer in the Neoadjuvant Chemotherapy Setting: A Retrospective Analysis. Ann. Surg. Oncol..

[19] Xu W., Jiang Y., Xu L., Li C., Wang J., Liu Z., Xue D., Gu Y., Zhong Z., He S. (2023). HER2-low status may predict poor neoadjuvant chemotherapy response in HR-negative breast cancer: A real-world multicenter study. Ultrasound Med. Biol..

[20] Ma Y., Zhu M., Zhang J., Lv M., Chen X., Liu Z. (2023). Prognostic Value of the Evolution of HER2-Low Expression after Neoadjuvant Chemotherapy. Cancer Res. Treat..

[21] Amin M.B., Greene F.L., Edge S.B., Compton C.C., Gershenwald J.E., Brookland R.K., Meyer L., Gress D.M., Byrd D.R., Winchester D.P. (2017). The Eighth Edition AJCC Cancer Staging Manual: Continuing to build a bridge from a population-based to a more “personalized” approach to cancer staging. CA Cancer J. Clin..

[22] Jeruss J.S., Mittendorf E.A., Tucker S.L., Gonzalez-Angulo A.M., Buchholz T.A., Sahin A.A., Cormier J.N., Buzdar A.U., Hortobagyi G.N., Hunt K.K. (2008). Combined use of clinical and pathologic staging variables to define outcomes for breast cancer patients treated with neoadjuvant therapy. J. Clin. Oncol..

[23] Mittendorf E.A., Vila J., Tucker S.L., Chavez-MacGregor M., Smith B.D., Symmans W.F., Sahin A.A., Hortobagyi G.N., Hunt K.K. (2016). The Neo-Bioscore Update for Staging Breast Cancer Treated with Neoadjuvant Chem-otherapy: Incorporation of Prognostic Biologic Factors Into Staging After Treatment. JAMA Oncol..

[24] Allison K.H., Hammond M.E.H., Dowsett M., McKernin S.E., Carey L.A., Fitzgibbons P.L., Hayes D.F., Lakhani S.R., Chavez-MacGregor M., Perlmutter J. (2020). Estrogen and Progesterone Receptor Testing in Breast Cancer: ASCO/CAP Guideline Update. J. Clin. Oncol..

[25] Wolff A.C., Hammond M.E.H., Allison K.H., Harvey B.E., Mangu P.B., Bartlett J.M.S., Bilous M., Ellis I.O., Fitzgibbons P., Hanna W. (2018). Human Epidermal Growth Factor Receptor 2 Testing in Breast Cancer: American Society of Clinical Oncology/College of American Pathologists Clinical Practice Guideline Focused Update. J. Clin. Oncol..

[26] Gradishar W.J., Moran M.S., Abraham J., Aft R., Agnese D., Allison K.H., Anderson B., Burstein H.J., Chew H., Dang C. (2022). NCCN clinical Practice guidelines in Oncology (NCCN Guidelines) breast cancer version 2.2023. J. Natl. Compr. Cancer Netw..

[27] Peiffer D.S., Zhao F., Chen N., Hahn O.M., Nanda R., Olopade O.I., Huo D., Howard F.M. (2023). Clinicopathologic Characteristics and Prognosis of ERBB2-Low Breast Cancer Among Patients in the National Cancer Database. JAMA Oncol..

[28] Gianni L., Eiermann W., Semiglazov V., Lluch A., Tjulandin S., Zambetti M., Moliterni A., Vazquez F., Byakhov M.J., Lichinitser M. (2014). Neoadjuvant and adjuvant trastuzumab in patients with HER2-positive locally advanced breast cancer (NOAH): Follow-up of a randomised controlled superiority trial with a parallel HER2-negative cohort. Lancet Oncol..

[29] Modi S., Jacot W., Yamashita T., Sohn J., Vidal M., Tokunaga E., Tsurutani J., Ueno N.T., Prat A., Chae Y.S. (2022). Trastuzumab Deruxtecan in Previously Treated HER2-Low Advanced Breast Cancer. N. Engl. J. Med..

[30] Schmid P., Cortés J., Marmé F., Rugo H.S., Tolaney S.M., Oliveira M., Loirat D., Jhaveri K., Yoon O.K., Motwani M. (2022). 214MO Sacituzumab govitecan (SG) efficacy in hormone receptor-positive/human epidermal growth factor receptor 2-negative (HR+/HER2–) metastatic breast cancer (MBC) by HER2 immunohistochemistry (IHC) status in the phase III TROPiCS-02 study. Ann. Oncol..

[31] Hurvitz S.A., Bardia A., Punie K., Kalinsky K., Cortés J., O’Shaughnessy J., Carey L., Rugo H., Yoon O., Pan Y. (2022). 168P Sacituzumab govitecan (SG) efficacy in patients with metastatic triple-negative breast cancer (mTNBC) by HER2 immunohistochemistry (IHC) status: Findings from the phase III ASCENT study. Ann. Oncol..

[32] Johnston S.R.D., Harbeck N., Hegg R., Toi M., Martin M., Shao Z.M., Zhang Q.Y., Rodriguez J.L.M., Campone M., Hamilton E. (2020). Abemaciclib Combined with Endocrine Therapy for the Adjuvant Treatment of HR+, HER2−, Node-Positive, High-Risk, Early Breast Cancer (monarchE). J. Clin. Oncol..

[33] Tutt A.N.J., Garber J.E., Kaufman B., Viale G., Fumagalli D., Rastogi P., Gelber R.D., de Azambuja E., Fielding A., Balmaña J. (2021). Adjuvant Olaparib for Patients with *BRCA1*- or *BRCA2*-Mutated Breast Cancer. N. Engl. J. Med..

[34] Masuda N., Lee S.-J., Ohtani S., Im Y.-H., Lee E.-S., Yokota I., Kuroi K., Im S.-A., Park B.-W., Kim S.-B. (2017). Adjuvant Capecitabine for Breast Cancer after Preoperative Chemotherapy. N. Engl. J. Med..

