# Supplementary material for: The Modified Neo-Bioscore System for Staging Breast Cancer Treated with Neoadjuvant Therapy Based on Prognostic Significance of HER2-Low Expression
Source: J Clin Med. 2024 Mar 23;13(7):1850. doi: 10.3390/jcm13071850 (PMC11012268; doi:10.3390/jcm13071850)
Supplement: Supplementary file 1 [file jcm-13-01850-s001.zip › jcm-2898058-supplementary.pdf]

Supplementary Online Content

Figure S1. Diagram of the study design

Figure S2. Classification of breast cancer based on different combinations of hormone receptor and HER2 status

Figure S3. Rates of pathological complete response ypT0 ypN0 (A) and ypT0/is ypN0 (B) by HER2 status in all the enrolled patients as well as subgroups by hormone receptor status

Figure S4. Kaplan-Meier survival analysis by HER2 status for locoregional relapse-free survival in the hormone receptor-positive patients (A) and overall survival in the hormone receptor-negative patients (B)

Figure S5. Multivariate Cox regression analysis for overall survival in all the enrolled patients

Figure S6. Receiver Operating Characteristic curve of Neo-Bioscore (A, C, E, G, I) and mNeo-Bioscore (B, D, F, H, J) for disease-free survival (A, B), relapse-free survival (C, D), locoregional relapse-free survival (E, F), distant relapse-free survival (G, H), and overall survival (I, J) in all the enrolled patients

Figure S7. Decision curve analysis of Neo-Bioscore and mNeo-Bioscore for disease-free survival (A), relapse-free survival (B), locoregional relapse-free survival (C), distant relapse-free survival (D), and overall survival (E) in all the enrolled patients

Table S1. Clinicopathological differences in patients with different HER2 status

Table S2. Changes on the number of patients according to different scoring principles of Neo-Bioscore and mNeo-Bioscore systems

Table S3. Akaike Information Criterion value of Neo-Bioscore and mNeo-Bioscore for disease-free survival, relapse-free survival, locoregional relapse-free survival, distant relapse-free survival, and overall survival in all the enrolled patients

Table S4. Concordance index value of Neo-Bioscore and mNeo-Bioscore for disease-free survival, relapse-free survival, locoregional relapse-free survival, distant relapse-free survival, and overall survival in all the enrolled patients

Table S5. Integrated Discrimination Improvement value of mNeo-Bioscore compared with Neo-Bioscore for disease-free survival, relapse-free survival,

locoregional relapse-free survival, distant relapse-free survival, and overall survival in all the enrolled patients during follow-up interval

Table S6. Five-Year overall survival rate by Neo-Bioscore and mNeo-Bioscore

This supplementary material has been provided by the authors to give readers additional information about their work.

Figure S1. Diagram of the study design.

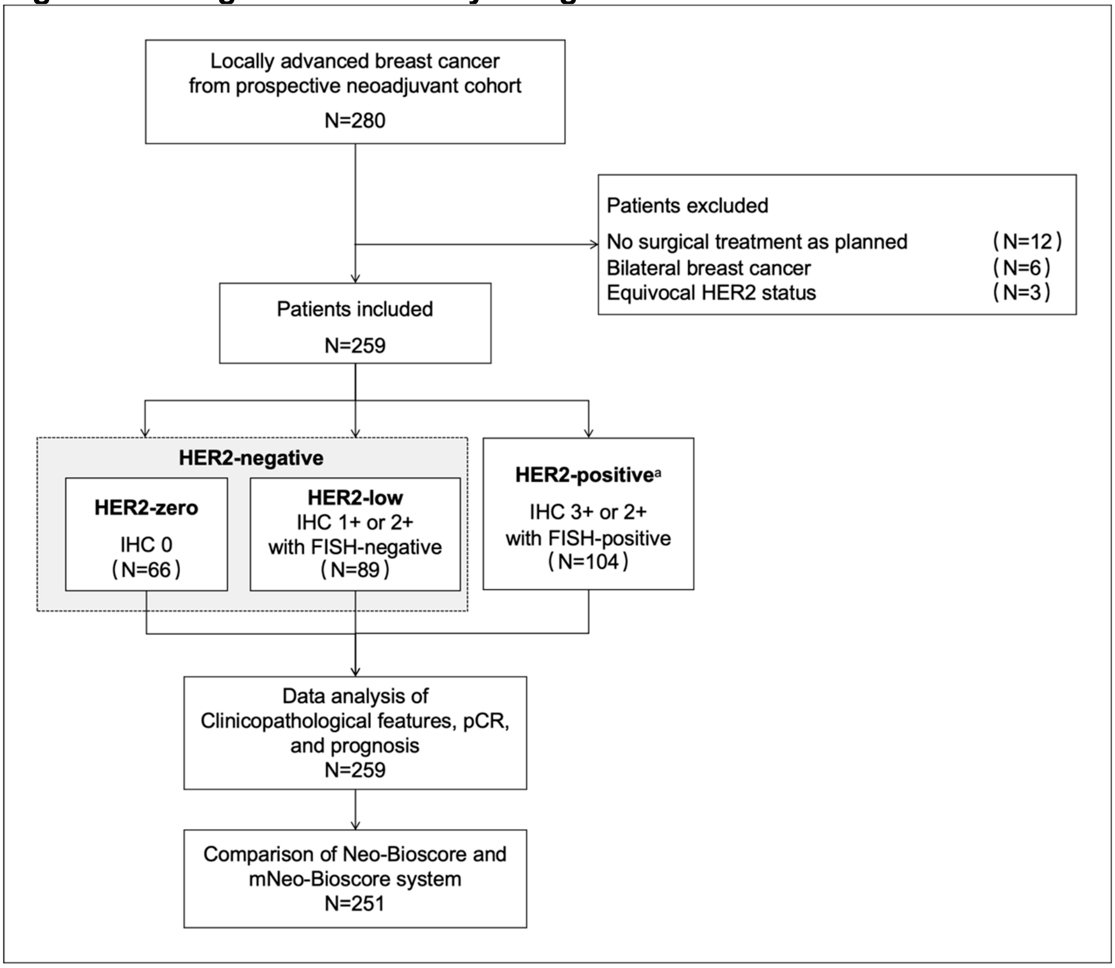

Abbreviations: HER2, human epidermal growth factor receptor 2; IHC, immunohistochemistry; FISH, fluorescent in situ hybridization; pCR, pathological complete response.

[a]HER2-positive patients were required to receive anti-HER2 target therapy concurrently.

Figure S2. Classification of breast cancer based on different combinations of hormone receptor and HER2 status.

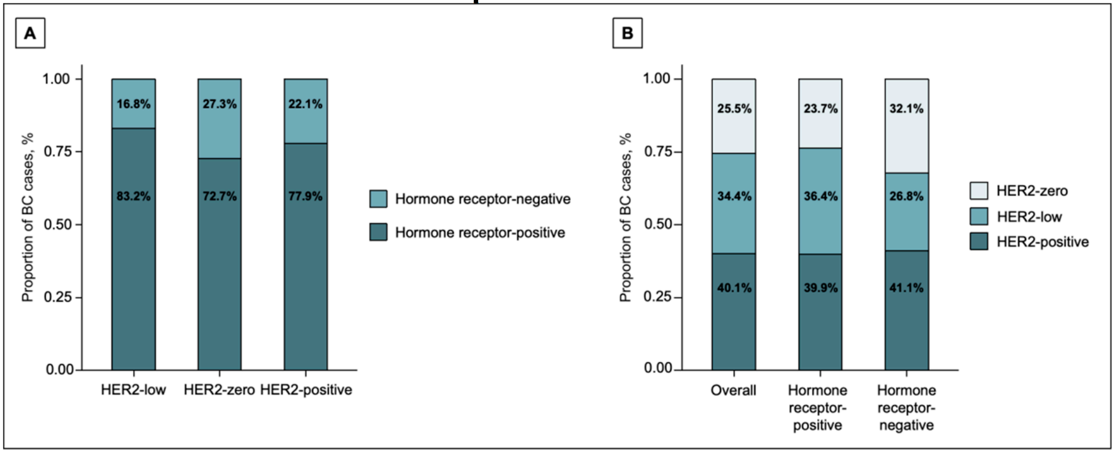

A. Prevalence of hormone receptor status categories for all the enrolled patients with different HER2 status; B. Prevalence of HER2 status categories for all the enrolled patients as well as subgroups by hormone receptor status.
Abbreviations: BC, breast cancer; HER2, human epidermal growth factor receptor 2.

Figure S3. Rates of pathological complete response ypT0 ypN0 (A) and ypT0/is ypN0 (B) by HER2 status in all the enrolled patients as well as subgroups by hormone receptor status.

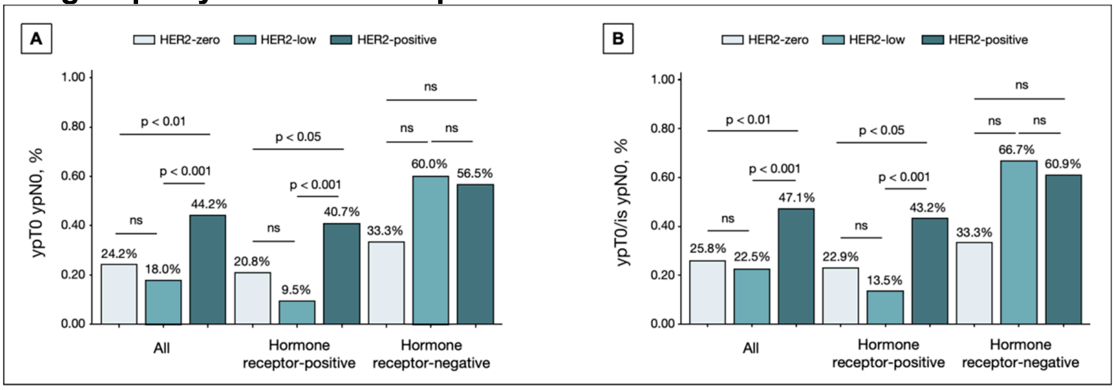

Abbreviations: HER2, human epidermal growth factor receptor 2; ns represents P > 0.05.

Figure S4. Kaplan-Meier survival analysis by HER2 status for locoregional relapse-free survival in the hormone receptor-positive patients (A) and overall survival in the hormone receptor-negative patients (B).

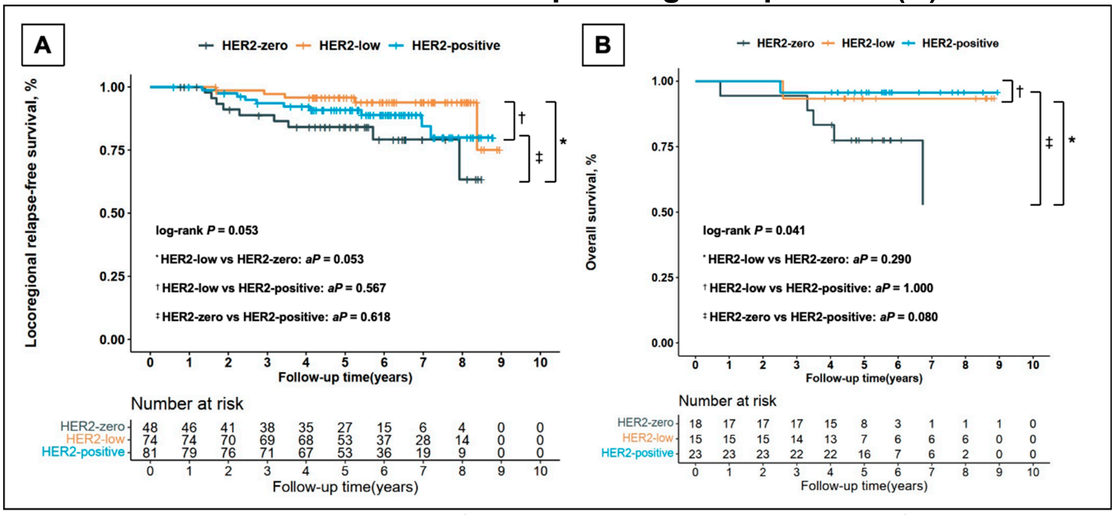

Abbreviations: HER2, human epidermal growth factor receptor 2; aP, P value adjusted by Bonferroni correction in all the enrolled patients.

Figure S5. Multivariate Cox regression analysis for overall survival in all the enrolled patients.

Characteristics	No. of Events / No. of Patients		Hazard Ratio (95% CI)	better worse	*P value*
**Age (years)**	**≤ 50**<br>7/115	**> 50**<br>9/136	0.989 (0.453 - 2.160)		0.978
**HER2 status**	**zero**<br>6/62	**low**<br>10/85	0.258 (0.087 - 0.765)		**0.015**
	**positive**<br>6/104	**low**<br>10/85	0.425 (0.146 - 1.230)		0.115
**Hormone receptor status**	**negative**<br>11/53	**positive**<br>5/198	0.817 (0.313 - 2.133)		0.680
**Ki-67 index**	**≤ 20**<br>3/61	**> 20**<br>13/190	3.259 (0.934 - 11.375)		0.064
**Histological grade**	**II**<br>5/105	**III**<br>11/146	1.437 (0.607 - 3.402)		0.410
**Clinical T stage**	**cT1 - cT3**<br>10/173	**cT4**<br>6/78	2.085 (0.927 - 4.690)		0.075
**Clinical N stage**	**cN0**<br>13/32	**cN1 - cN3**<br>3/219	0.433 (0.153 - 1.222)		0.114
**Pathological T stage**	**ypT0 - ypT1**<br>12/211	**ypT2 - ypT3**<br>4/40	1.768 (0.713 - 4.388)		0.219
**Pathological N stage**	**ypN0**<br>10/147	**ypN1 - ypN3**<br>6/107	3.337 (1.376 - 8.093)		**0.008**

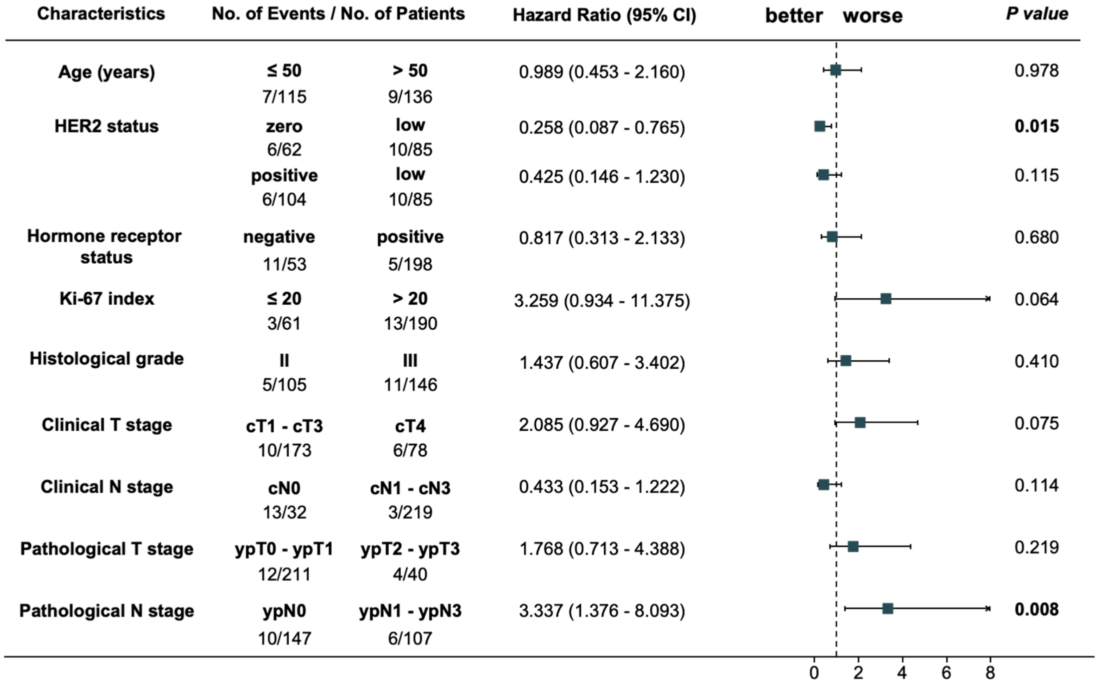

Abbreviations: HER2, human epidermal growth factor receptor 2; CI, confidence interval.

Figure S6. Receiver Operating Characteristic curve of Neo-Bioscore (A, C, E, G, I) and mNeo-Bioscore (B, D, F, H, J) for disease-free survival (A, B), relapse-free survival (C, D), locoregional relapse-free survival (E, F), distant relapse-free survival (G, H), and overall survival (I, J) in all the enrolled patients.

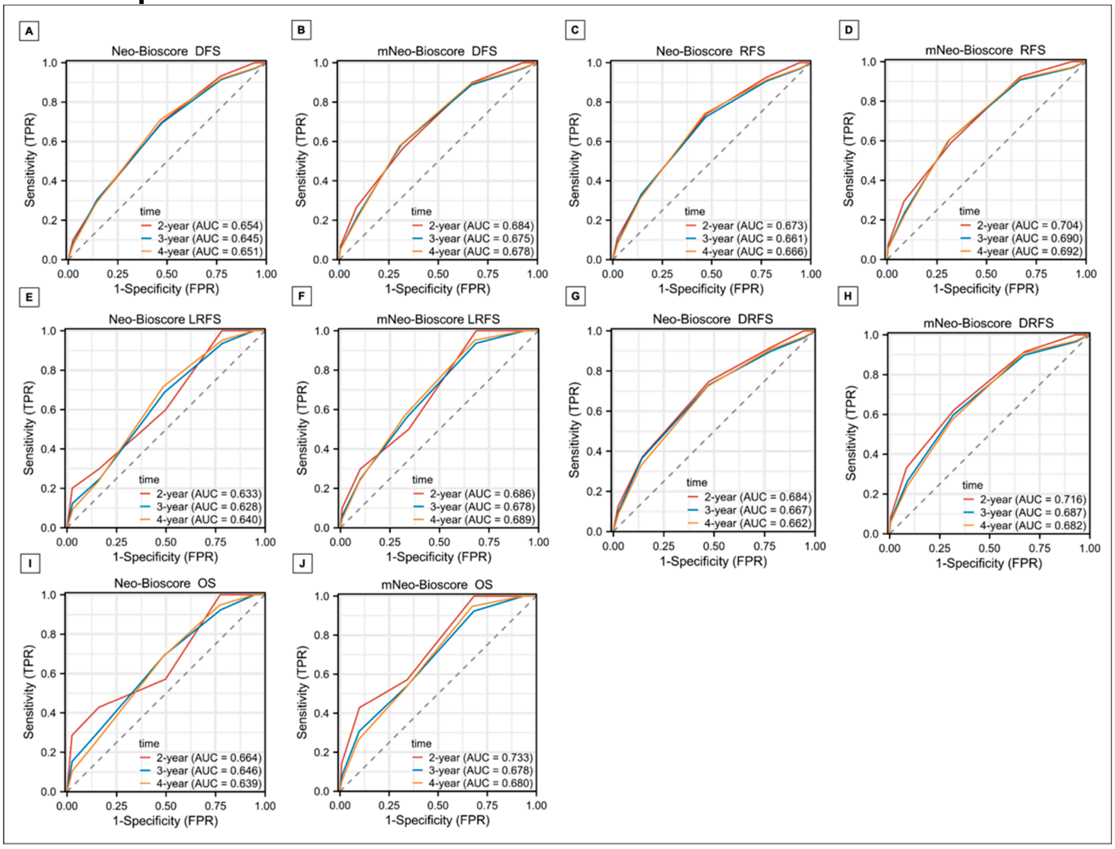

Figure S7. Decision curve analysis of Neo-Bioscore and mNeo-Bioscore for disease-free survival (A), relapse-free survival (B), locoregional relapse-free survival (C), distant relapse-free survival (D), and overall survival (E) in all the enrolled patients.

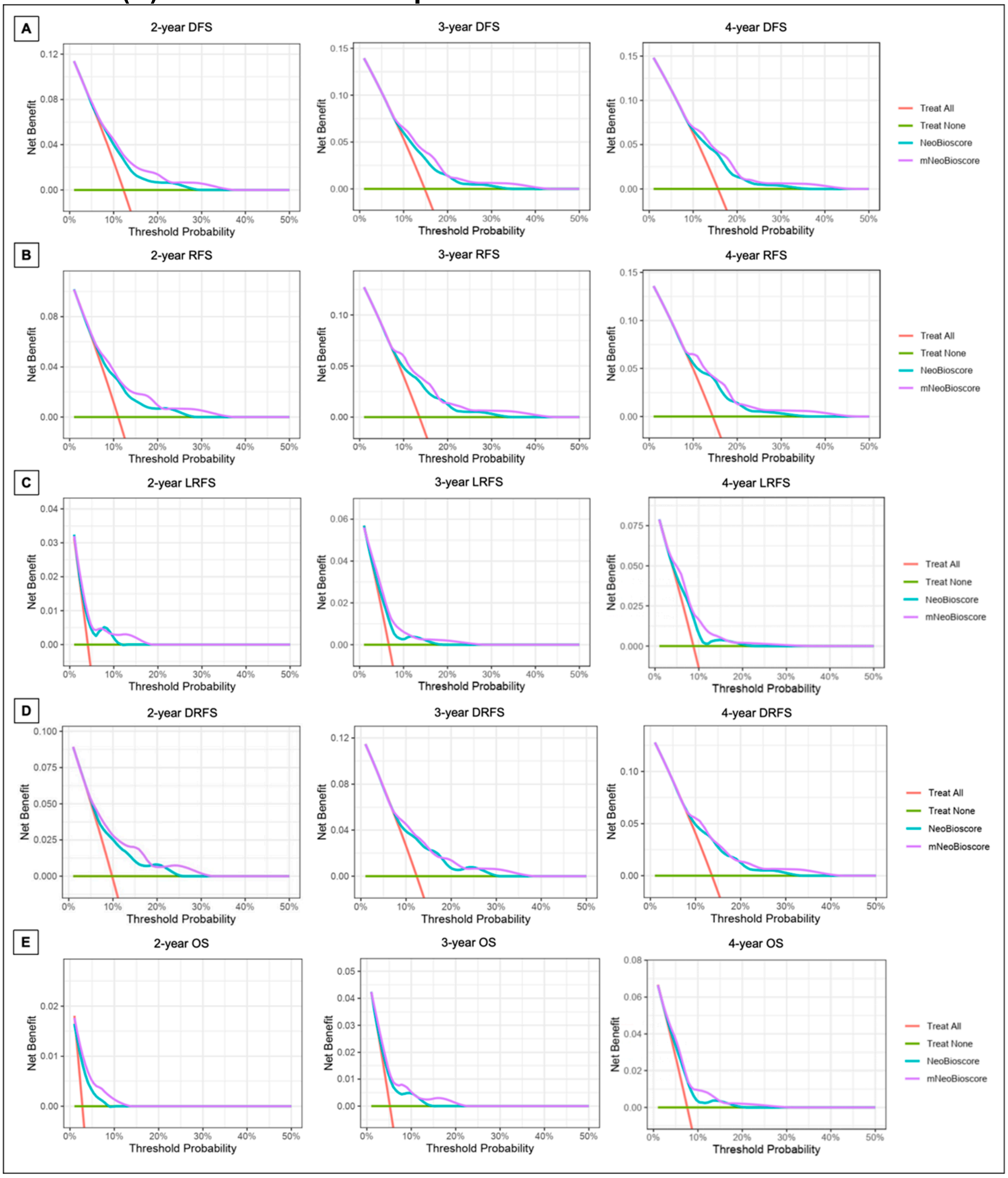

Table S1. Clinicopathological differences in patients with different HER2 status.

	HER2-zero (n=66)	HER2-low (n=89)	HER2-positive (n=104)	*P* value			
				all patients	low vs zero	low vs positive	zero vs positive
**Age，years**							
median (IQR)	48 (41, 60)	52 (43, 59)	53 (46, 60)	0.180	0.656	0.142	0.085
>50	28 (42.4%)	47 (52.8%)	63 (60.6%)	0.069	0.201	0.277	0.021
≤50	38 (57.6%)	42 (47.2%)	41 (39.4%)				
**Hormone receptor status**							
Negative	18 (27.3%)	15 (16.9%)	23 (22.1%)	0.293	0.117	0.360	0.444
Positive	48 (72.7%)	74 (83.1%)	81 (77.9%)				
**Ki-67 index**							
median (IQR)	50 (30, 70)	30 (20, 50)	40 (30, 60)	**0.010**	**0.006**	**0.029**	0.188
>20%	53 (80.3%)	59 (66.3%)	85 (81.7%)	**0.028**	**0.054**	**0.014**	0.816
≤20%	13 (19.7%)	30 (33.7%)	19 (18.3%)				
**Histological grade**							
Grade II	30 (48.4%)	44 (51.8%)	31 (29.8%)	**0.005**	0.686	**0.002**	0.016
Grade III	32 (51.6%)	41 (48.2%)	73 (70.2%)				
NA[a]	4	4	0				
**Clinical T stage**							
cT1	0 (0%)	0 (0%)	2 (1.9%)	0.451	0.264	0.590	0.258
cT2	15 (22.7%)	17 (19.1%)	22 (21.1%)				
cT3	36 (54.6%)	41 (46.1%)	45 (43.3%)				
cT4	15 (22.7%)	31 (34.8%)	35 (33.7%)				
**Clinical N stage**							
cN0	9 (13.6%)	14 (15.7%)	13 (12.5%)	0.671	0.899	0.580	0.315

cN1	45 (68.2%)	62 (69.7%)	79 (76.0%)				
cN2	2 (3.0%)	3 (3.4%)	5 (4.8%)				
cN3	10 (15.2%)	10 (11.2%)	7 (6.7%)				
**Clinical staging**							
IIA	1 (1.5%)	1 (1.1%)	3 (2.9%)				
IIB	18 (27.3%)	23 (25.9%)	29 (27.9%)				
IIIA	25 (37.9%)	29 (32.6%)	33 (31.7%)	0.552	0.607	0.832	0.191
IIIB	12 (18.2%)	26 (29.2%)	32 (30.8%)				
IIIC	10 (15.1%)	10 (11.2%)	7 (6.7%)				
**Pathological T stage**							
ypT0	24 (36.4%)	29 (32.6%)	63 (60.6%)				
ypTis	1 (1.5%)	4 (4.5%)	3 (2.9%)				
ypT1	31 (47.0%)	36 (40.5%)	27 (26.0%)	**0.005**	0.580	**0.002**	**0.025**
ypT2	9 (13.6%)	19 (21.3%)	9 (8.6%)				
ypT3	1 (1.5%)	1 (1.1%)	2 (1.9%)				
**Pathological N stage**							
ypN0	33 (50.0%)	37 (41.6%)	78 (75.0%)				
ypN1	18 (27.3%)	27 (30.3%)	15 (14.4%)	**< 0.001**	0.493	**< 0.001**	**0.002**
ypN2	5 (7.6%)	13 (14.6%)	8 (7.7%)				
ypN3	10 (15.1%)	12 (13.5%)	3 (2.9%)				
**Pathological staging**							
0	17 (25.8%)	20 (22.5%)	49 (47.1%)	**< 0.001**	0.529	**< 0.001**	**0.003**
I A	12 (18.2%)	11 (12.3%)	26 (25.0%)				

II A	19 (28.8%)	25 (28.1%)	15 (14.4%)
II B	2 (3.0%)	8 (9.0%)	3 (2.9%)
III A	6 (9.1%)	13 (14.6%)	8 (7.7%)
III C	10 (15.1%)	12 (13.5%)	3 (2.9%)

Abbreviations: HER2, human epidermal growth factor receptor 2; IQR, Interquartile Range; NA, not applicable. [a]Histological grade could not be assessed in 8 patients.

Table S2. Changes on the number of patients according to different scoring principles of Neo-Bioscore and mNeo-Bioscore systems.

Score	**Neo-Bioscore**	**mNeo-Bioscore**
0	0	1
1	12	14
2	43	62
3	68	83
4	82	60
5	33	23
6	7	2
7	1	1

Table S3. Akaike Information Criterion value of Neo-Bioscore and mNeo-Bioscore for disease-free survival, relapse-free survival, locoregional relapse-free survival, distant relapse-free survival, and overall survival in all the enrolled patients.

Survival outcome	Neo-Bioscore	mNeo-Bioscore
**DFS**	523.79	522.09
**RFS**	491.46	489.62
**LRFS**	299.52	295.69
**DRFS**	469.65	468.37
**OS**	280.39	277.4

Table S4. Concordance index value of Neo-Bioscore and mNeo-Bioscore for disease-free survival, relapse-free survival, locoregional relapse-free survival, distant relapse-free survival, and overall survival in all the enrolled patients.

Survival outcome	Neo-Bioscore	mNeo-Bioscore
**DFS**	0.641 (0.547~0.736)[a]	0.662 (0.568~0.756)
**RFS**	0.656 (0.558~0.753)	0.676 (0.582~0.770)
**LRFS**	0.653 (0.531~0.774)	0.708 (0.601~0.816)
**DRFS**	0.649 (0.545~0.752)	0.667 (0.567~0.767)
**OS**	0.646 (0.514~0.777)	0.697 (0.580~0.815)

Data were presented as concordance index (95% confidence interval).

Table S5. Integrated Discrimination Improvement value of mNeo-Bioscore compared with Neo-Bioscore for disease-free survival, relapse-free survival, locoregional relapse-free survival, distant relapse-free survival, and overall survival in all the enrolled patients during follow-up interval.

**Survival outcome**	2nd year	3rd year	4th year
**DFS**	0.001	0.001	0.001
**RFS**	0.012	0.012	0.012
**LRFS**	0.008	0.012	0.015
**DRFS**	0.011	0.009	0.009
**OS**	0.008	0.009	0.012

Table S6. Five-Year overall survival rate by Neo-Bioscore and mNeo-Bioscore.

Neo-Bioscore	5-year OS rate (95% CI), %	mNeo-Bioscore	5-year OS rate (95% CI), %
0 (n=0)		0 (n=1)	100
1 (n=12)	100	1 (n=14)	100
2 (n=43)	94.93 (81.15-98.71)	2 (n=62)	96.50 (86.67-99.12)
3 (n=68)	92.89 (83.75-96.98)	3 (n=83)	90.70 (82.26-95.24)
4 (n=82)	90.36 (81.65-95.06)	4 (n=60)	91.64 (81.07-96.43)
5 (n=33)	91.08 (74.83-97.03)	5 (n=23)	83.23 (61.27-93.36)
6-7 (n=8)	61.36 (21.40-85.67)	6-7 (n=3)	33.33 (9-77.41)

Abbreviations: OS, overall survival; CI, confidence interval.
